# Alcohol use and the pain system

**DOI:** 10.3389/adar.2024.12005

**Published:** 2024-01-24

**Authors:** Michael Vigorito, Sulie L. Chang

**Affiliations:** ^1^ Institute of NeuroImmune Pharmacology, Seton Hall University, South Orange, NJ, United States; ^2^ Department of Biological Sciences, Seton Hall University, South Orange, NJ, United States

**Keywords:** alcohol misuse, nociception, pain-associated alcohol dependence, neuroimmune interaction, pain pathways, c-FOS, hyperkatifeia

## Abstract

The World Health Organization’s epidemiological data from 2016 revealed that while 57% of the global population aged 15 years or older had abstained from drinking alcohol in the previous year, more than half of the population in the Americas, Europe, and Western Pacific consumed alcohol. The spectrum of alcohol use behavior is broad: low-risk use (sensible and in moderation), at-risk use (e.g., binge drinking), harmful use (misuse) and dependence (alcoholism; addiction; alcohol use disorder). The at-risk use and misuse of alcohol is associated with the transition to dependence, as well as many damaging health outcomes and preventable causes of premature death. Recent conceptualizations of alcohol dependence posit that the subjective experience of pain may be a significant contributing factor in the transition across the spectrum of alcohol use behavior. This narrative review summarizes the effects of alcohol at all levels of the pain system. The pain system includes nociceptors as sensory indicators of potentially dangerous stimuli and tissue damage (nociception), spinal circuits mediating defensive reflexes, and most importantly, the supraspinal circuits mediating nocifensive behaviors and the perception of pain. Although the functional importance of pain is to protect from injury and further or future damage, chronic pain may emerge despite the recovery from, and absence of, biological damage (i.e., in the absence of nociception). Like other biological perceptual systems, pain is a construction contingent on sensory information and a history of individual experiences (i.e., learning and memory). Neuroadaptations and brain plasticity underlying learning and memory and other basic physiological functions can also result in pathological conditions such as chronic pain and addiction. Moreover, the negative affective/emotional aspect of pain perception provides embodied and motivational components that may play a substantial role in the transition from alcohol use to dependence.

## Introduction

In recognition of the diversity and complexity of pain revealed by recent clinical and basic science, the *International Association for the Study of Pain* (IASP) re-evaluated their widely adopted definition of pain and revised it to “An unpleasant sensory and emotional experience associated with, or resembling that associated with, actual or potential tissue damage.” The difficulty of encompassing all aspects of pain in a single definition necessitated the inclusion of 6 bulleted notes for further consideration [1]. In this narrative review of the pain system and alcohol use we incorporate the IASP definition and notes and make the following basic distinctions. The sensation of pain is the subjective (conscious) experience of pain in response to the biological detection of dangerous or potentially dangerous stimuli. The sensation of pain is referred to as *nociceptive-pain* rather than nociception since nociception and pain are related but not identical constructs. In contrast, the perception of pain is a subjective experience that is primarily a psychological process involving the brain’s systematic analysis and interpretation of physical information concerning potentially dangerous stimuli (including nociceptive-pain) and tissue damage. The perception of pain, which from here on will be referred to simply as pain, is challenging to study because it involves biological, psychological, and social factors and is learned through life experiences.

Pain can function adaptively in the short term (acute pain) and long term (e.g., by inducing learned behavioral change) or maladaptively in a chronic manner. Chronic pain is not a single maladaptive entity but reflects a progression from different pathologies. N*europathic pain*, for example, which requires an injury diagnosis such as nerve trauma or stroke, emerges from adaptive changes that lead to a chronic painful syndrome. *Inflammatory pain* is an adaptive response that sensitizes a nociceptive neural circuit to increase nociceptive-pain, but dysfunction in this adaptive response is a likely contributor to the transition from acute to chronic pain conditions [2]. The upregulation of nociceptor ion channels induces spontaneous activity causing a persistent nociceptive-pain experience that motivates recuperative behavior. This *sensitization* of nociceptors results in increased sensitivity at the site of exposure to the noxious stimulus (*primary hyperalgesia*) and to the surrounding area (*secondary hyperalgesia*) and can also induce the sensation of pain from thermal or mechanical stimuli that are normally innocuous (i.e., *allodynia*) [3, 4]. Many other inflammatory signals also impact on nociceptors as downstream targets by inducing upregulation of ion channels including histamine, bradykinins, prostaglandin E2, nerve growth factor (NGF), and protons H+. Hypersensitivity of neural circuitry also occurs in the spinal and supraspinal circuits of the central nervous system (CNS) and by consensus is conceptualized as *central sensitization* [2, 5].

Pain is considered chronic when it persists or recurs beyond a usual recovery period of about 3–6 months or when associated with a chronic health condition (e.g., cancer) [6]. Because pain is a subjective and emotional response to a personal experience, reliable self-report measures are the best indicators of a person’s pain experience. But as noted by the IASP council, the inability to communicate an expression of pain does not indicate the absence of pain in human or non-human animals. Several strategies are used to assess patients who are unable to self-report [7]. Measures of pain-like behaviors have been developed in preclinical animal models of nociceptive-pain and chronic pain [8].

## Nociception and nociceptive-pain

Traditionally, nociception refers to the sensing of noxious (intense) stimuli impinging on the body from the external (e.g., skin) or internal (e.g., muscles, viscera) environment by the class of sensory neurons named “noci-ceptors” by Sir Charles Sherrington [9]. Nociceptors expressed by first-order sensory neurons of the spinal cord (dorsal root ganglion, DRG), for example, transduce signals—mechanical, thermal, or chemical—from the environment into neural information that is conducted to second-order neurons within the dorsal horn of the spinal cord for nociceptive processing. The relay of nociceptive information to the brain is necessary for the subjective (conscious) sensation of pain (i.e., nociceptive-pain), however nociception itself (i.e., nociceptor activity) is not sufficient for the sensation of pain nor necessary for the perception of pain. Indeed, Sherrington introduced the concept of nociception to account for the skin’s “special sense of its own injury” and the discovery, in an experimental spinal dog preparation, that a reflexive defensive withdrawal response continues to be elicited despite the separation of the spinal cord from the brain [10]. The dissociation of nociception from the sensation of pain is also evident in non-experimental contexts. For example, cough is a nociceptor-driven response that is not typically accompanied with nociceptive-pain [11, 12]. Nociceptor activation also plays a role in the protection against muscle injury under normal behavior repertoires by triggering innate motor patterns through spinopallidal circuits independent of the neural circuitry necessary for the cognitive or affective components of pain [13].

In animals, nociception and nociceptive-pain are assessed and inferred, respectively, using several accepted stimulus-dependent tests (see [8]). For humans, the *nociceptive flexion reflex* (NFR) is a popular objective neurophysiological tool for the assessment of nociception and nociceptive-pain. This polysynaptic reflex is activated involuntarily by noxious stimuli applied to a limb causing a protective withdrawal response. Because the NFR is moderately positively correlated with verbal reports of pain this measure is also used as an indicator of nociceptive-pain [14]. However, there are reports of the dissociation between the NFR and nociceptive-pain under clinically relevant (e.g., chronic pain syndromes) and normal situations [15–17]. It has also been shown under experimental contexts that stimulus-dependent withdrawal reflexes are influenced by cognitive and emotional factors modulating descending control of spinal circuits [18].

Our understanding of nociception as a defensive bodily response that is separate, although often concurrent with, nociceptive-pain has expanded remarkably by findings that nociceptors engage the immune system directly in defensive barrier functions and disturbances in homeostasis [19, 20]. A recent study utilizing newly developed optoelectronic technology confirms that nociceptor activation is sufficient to directly induce activation of innate and acquired immune cells [21]. The role of direct neural activation of immune function in response to physical insult, known as *neurogenic inflammation*, has long been recognized [22–24]. A well-established mediator of neurogenic inflammation are nociceptors that release calcitonin gene-related peptide (CGRP) and substance P antidromically to induce endothelial and smooth muscle cells to produce vasodilation, increased vascular permeability, and edema, resulting in the experience of redness, heat, and swelling at the site of injury. As first noted at the start of the first millennium A.D. by the Roman encyclopedist Aulus Cornelius Celsus, nociceptive-pain (dolor) accompanies the other three symptoms of inflammation -rubor, calor and tumis, respectively [25]. However, even when nociception is experienced as a sensation (i.e., nociceptive-pain), it is not just a symptom of bodily harm. Nociceptors are actively engaged in the regulation of inflammation by sensing pathogens and contributing to inflammation and the subsequent recovery of homeostasis.

Neuro-immune interaction in defensive action, homeostatic recovery, and maintenance is incompletely understood. For example, a recent study calls attention to our gaps in understanding of neuroimmune processes in the treatment of acute pain and the transition of acute pain to chronic pain. Treatment with steroidal and non-steroidal anti-inflammatory drugs for early musculoskeletal pain conditions have hypoalgesic efficacy, however early anti-inflammatory treatment interfered with a protective effect of acute inflammatory responses against the development of chronic pain in the long-term [26]. As is discussed below, a similar paradoxical effect is seen with alcohol. In animals and humans acute alcohol consumption has hypoalgesic properties,^1^ but when alcohol consumption transitions to chronic consumption it hastens the progression to chronic pain a condition that is highly comorbid with alcohol misuse and Alcohol Use Disorder (AUD) [27]. A spotlight on the impact of different degrees of alcohol consumption on nociception, nociceptive-pain, and chronic pain may yield insight into neuroinflammatory processes and chronic pain and their role in the development and maintenance of alcohol misuse and AUD [28].

### Molecular aspects of nociception

Nociceptors that result in intense short-term nociceptive-pain (sometimes referred to as “primary pain”) are fast-acting myelinated (Aδ) neurons. Slow unmyelinated neurons (C) transmitting diffuse signals are experienced as dull, prolonged nociceptive-pain. The primary neurotransmitter is the excitatory neurotransmitter glutamate, but nociceptors are also modulated by several endogenous peptides at their peripheral and central terminals [29]. The molecular mechanisms of nociceptors are highly heterogenous. Nociceptors express many ion channels including specialized voltage-gated sodium channels (Na_v_1.7, Na_v_1.8, and Na_v_1.9), mechanosensitive Piezo ion channels (Piezo1, Piezo2) and the transient receptor potential (TRP) channels. Among the latter are the TRP vallinoid 1 (TRPV1) and TRP ankyrind 1 (TRPA1) ligand-gated channels, also known as the capsacin receptor and the wasabi receptor, respectively. In addition to the sensing of mechanical (Peizo), temperature (TRPV1), and chemical (TRPA1) *danger* signals, nociceptors also detect *damage*-associated molecular patterns (DAMP) released from damaged tissue. DAMPS bind to pattern-recognition receptors such as Toll-like receptors (TLRs 3, 4, 7 and 9), signaling the innate immune system to promote a non-infectious inflammatory response [19]. For example, the chromatin-associated protein HMGB1 (high mobility group box 1) when secreted into the extracellular environment functions as an inflammatory cytokine. The binding of HMGB1 to TLR4 generates reactive oxygen species (ROS) and the downstream activation of nuclear factor kappa-light-chain-enhancer of activated B cells (NF-κB) to induce proinflammatory gene activation [30, 31]. HMGB1 activation of NF-κB and ROS generation is also mediated through the stimulation of the receptor for advanced glycation end products (RAGE). Another major DAMP is ATP (adenosine triphosphate) detected by immune cells and nociceptors because of the expression of purinergic receptors on both cell types. Alcohol contributes to peripheral and central pain processing by directly inducing the release of DAMPS as a result of the toxic effects of the alcohol degradation product acetaldehyde and its byproducts or by impacting on DAMP mediated inflammatory reactions induced by other physical damages [32].

In addition to DAMPS neuro-immune interaction may be disrupted by gut-derived pathogens [22, 33]. Nociceptors detect microbial pathogens through pathogen-associated molecular patterns (PAMP) (e.g., LPS, flagellin, peptidoglycans) and bacterial products (e.g., N-Formyl peptides). Although gut microbiota is well established as a modulator of visceral pain, substantial evidence is accumulating that gut microbiota also play a role in many types of chronic pain, including inflammatory and neuropathic pain, by impacting on the peripheral and central nervous system [34].

The ability of nociceptors to detect pathogens and modulate the experience of pain through bidirectional neuroimmune integration reflects the broader ability of sensory neurons to interact with the microbiome, including symbiotic (or commensal) microbiota to form a microbiota-gut-brain axis (for review see [35]). A role of symbiotic microbes in the causal mediation of nociceptive-pain has been confirmed by the experimental construction of axenic or “germ-free” mice made free from all microorganisms by preventing natural colonization by microorganisms. Behavioral measures of nociception in germ-free mice indicated reduced nociceptor sensitization to experimentally induced inflammatory signals which was reversed with restoration of microbiota using fecal transplants from conventional mice. Additionally, the commensal microbiota may have restored nociceptor sensitization by stimulating toll-like receptors [36].

Nociceptors are also modulated by the immune cells of the innate and acquired immune systems under pathological conditions resulting from tissue injury and infection by responding to molecular modulators including cytokines (tumor necrosis factors [TNF], interleukins (IL), interferons [IFN], chemokines [e.g., CCL1, CCL2], transforming growth factor [TGF], and prostaglandins (e.g., PGE2) [37]. These molecular modulators of nociceptive processing occur at all levels of the pain system including the peripheral nervous system (peripheral nociceptor terminals, dorsal root ganglion) and central nervous system (spinal cord, supraspinal brain circuits) [38]. Alcohol can alter these processes by producing dysbiosis of the gut microbiome which then impacts on peripheral nociceptors and the gut-brain communication through several pathways including through the vagus nerve [39, 40].

It is important to note that most of these studies, as with studies on pain and alcohol use and dependence in general, have been conducted with male subjects. More recent evidence, although limited, provide compelling evidence that there are sex differences in neuroimmune signaling and synaptic function as well as the disruptions that occur following chronic alcohol consumption. Sex differences can be seen in studies on transcriptomic analyses, cytokine regulation of the innate and acquired immune system, and regulation of alcohol intake by astrocytes and microglia (for a detailed review see [41]). Research on biological sex-dependent neuroimmune mechanisms is likely to provide insight into the relationship between gender and pain such as why woman have more experiences with perceived acute pain and show greater prevalence of some forms of chronic pain (e.g., fibromyalgia) [42]. Moreover, changes during aging in pain sensitivity, chronic pain, and the role of molecular mechanisms including via neuroinflammation is still not well characterized [43].

### Alcohol, nociception, and nociceptive-pain

Numerous experiments in animals convincingly demonstrate that forced administration or voluntary consumption of alcohol has short-term hypoalgesic properties as indicated by raised nociceptive thresholds in response to thermal stimuli (tail flick and hot-plate tests) and other measures of nociceptive-pain and allodynia (e.g., Von Frey mechanical sensitivity) (for review of different methods to measure nociceptive-pain in rodents see [44] and for the effects of alcohol on these measures see [45]). For example, Gatch and Lal [46] showed that alcohol administered to rats acutely (i.p.) induces hypoalgesia (dose-dependently) and when given chronically in a liquid diet. Although the hypoalgesic effect of chronic alcohol shows tolerance, withdrawal of alcohol induces hyperalgesia that is reversed by re-administration of alcohol. Withdrawal-induced hyperalgesia and mechanical allodynia is also seen when alcohol is given as a chronic intermittent ethanol vapor although the effects are moderated by several factors including amount of alcohol exposure and sex [47–49]. Protocols using intermittent chronic alcohol exposure in rodents have been used successfully as reliable and valid animal models of drug and alcohol dependence. Preclinical studies on chronic pain and AUD provide new insight into the reciprocal influences between the common morbidity of pain and alcohol dependence and potential treatment strategies [45].

Alcohol can also have robust dose-dependent analgesic properties in healthy human volunteers experiencing experimentally induced nociceptive-pain [50, 51]. Although experimental nociceptive-pain differs in many ways with clinical pain, there is evidence that the analgesic properties of alcohol may support self-medication behaviors of pain sufferers. Experimental induction of a moderate but clinically significant acute pain (capsaicin plus heat) increased the urge and intention to drink alcohol in healthy undergraduate students reporting frequent drinking experiences [52]. Several studies have reported an association between moderate alcohol use and reduced pain especially in men [51, 53, 54]. A recent ecologically relevant experimental study investigating behavioral economic measures of the self-medicating use of alcohol following induced delayed musculoskeletal pain (i.e., a common experience of delayed onset muscle soreness that occurs after exertion) revealed an increased demand for alcohol in males, although a decreased demand in women [55]. The hypoalgesic effects of alcohol consumption can also be observed despite the presence of chronic pain [56]. Paradoxically, as discussed further below, alcohol may be an effective hypoalgesic for the short-term relief of pain but long-term consumption of alcohol results in exacerbated pain, increasing an individual’s risk towards alcohol misuse and the development of AUD [51]. The sex specific effects in these studies support existing research highlighting sex (biological) and gender (psychosocial) differences in pain perception and tolerance [41, 57] and suggest that men are at increased risk of developing AUD when self-medicating for nociceptive-pain, despite many studies indicating that females are disproportionally affected by chronic pain [58].

### Neuroimmune interactions and alcohol

The regulation of the immune system is intricate and made even more complex by its bidirectional communication with the nervous system. The complexity of alcohol’s modulation of these functions reveals itself in paradoxical ways. It is clear that alcohol modulates innate immunity to microbial products in a dose- and time-dependent manner, although the relationship among these variables is inconsistent in the literature most likely due to differences in methodology and parameters. While most studies are based on *in vitro* experiments, *in vivo* studies confirm opposing effects of alcohol exposure on the inflammatory response of innate immune cells. For example, while short-term exposure (hours) reduces levels of systemic inflammation, long–term exposure (days) stimulates proinflammatory cytokines and decreases anti-inflammatory cytokines [59]. Similar temporal differences of drinking on immune function may explain observations that light to moderate drinking improves responses to vaccines, but heavy chronic drinking is associated with immune dysfunction [60]. It is unclear if alcohol’s hypoalgesic effects in short-term drinking and hyperalgesia in chronic drinkers reflect this paradoxical effect of alcohol on immune function.

The initial impact of alcohol following its consumption is of course on the gastrointestinal system, being absorbed mainly in the upper intestines and entering the blood circulation and the portal circulation to the liver. The presence of ethanol in the blood also serves to maintain persistent levels of alcohol throughout the gastrointestinal tract until alcohol is eliminated through several metabolic pathways. The most relevant pathway in light to moderate drinkers is the metabolism by alcohol dehydrogenase (ADH) in the liver into the toxic compound acetaldehyde potentially causing hepatocyte injury and the release of DAMPS. Aldehyde dehydrogenase (ALDH) then metabolizes acetate into a less toxic compound, which is then metabolized to Acetyl CoA, a product that is also a key metabolite of the major nutrients—carbohydrates, fat, and protein. Another pathway, especially in heavy drinkers, is Cytochrome P450 2E1 (CYP2E1) which results in ROS contributing to oxidative stress [32, 61]. Studies in humans and animals demonstrate that in the presence of chronic alcohol exposure there are increases in bacterial loads and in the permeability of the gastrointestinal barrier allowing bacteria of the microbiome and their endotoxins (i.e., lipopolysaccharides, LPS) to enter the bloodstream [62]. Preclinical studies with rodents show that a “leaky gut” due to repeated cycles of alcohol exposure increases the release of LPS which affects peripheral and brain immune (i.e., microglia) signaling that may also lead to the progression and persistence of problematic alcohol use behavior [30, 39]. As discussed later, the role of pain in alcohol misuse and AUD has become an important area of interest [63]. Because LPS also acts directly on TRPA1 channel of nociceptors to induce a rapid modulation of nociception and nociceptive-pain, a “leaky gut” may contribute to the progression and maintenance of maladaptive alcohol use by modulating alcohol-associated nociception, nociceptive-pain, and chronic pain [64]. This possibility needs to be further investigated.

Thus, nociceptor component of the pain system and the immune system share the role of detecting acute perturbations in homeostasis due to noxious stimuli and potentially pathogenic microbes and engage in integrated protective countermeasures—from adjustments in behavior (to minimize tissue injury and to escape and avoid dangerous stimuli) to the neutralization of pathogens, resolution of inflammation, and the restoration of tissue homeostasis. Nevertheless, given that the neuron-immune integration to dangerous and damaging stimuli is varied and extremely complex, it is not surprising that these processes can become dysfunctional leading to failed or maladaptive homeostasis resulting in disease processes such as chronic pain. The role of alcohol in these neuro-immune processes as it relates to pain is understudied. Furthermore, nociception needs to be viewed more broadly, not simply as the direct initiator of nociceptive-pain and the perception of pain but in a broader context of neuro-immune regulation and possible alcohol-induced dysfunction of homeostasis and allostasis.

## Spinal and supraspinal circuit structures

**FIGURE 1:**
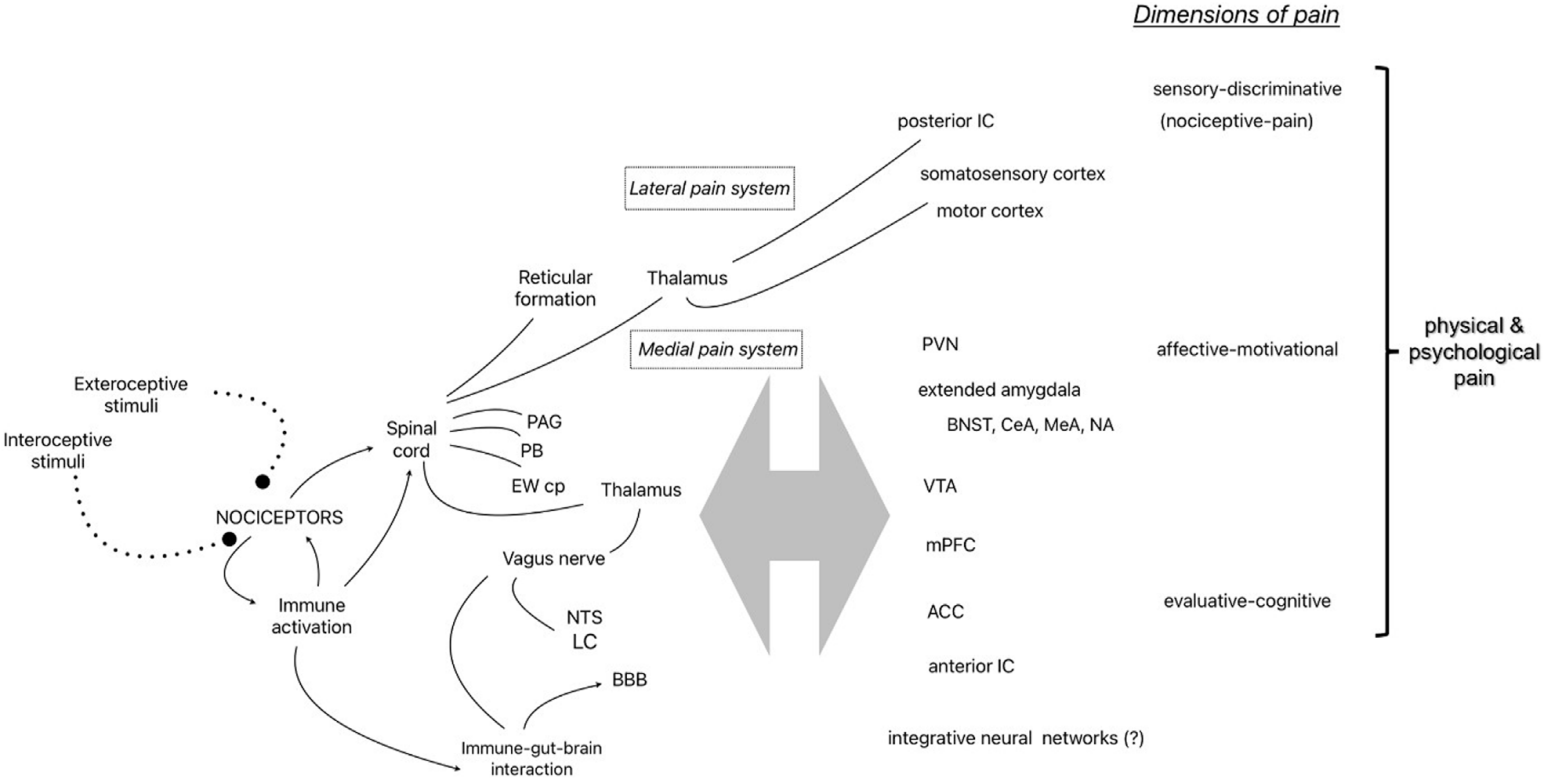
Representative components of the pain system identified in the narrative review and the 3 dimensions of physical and psychological pain [55]. Abbreviations: PAG, periaqueductal Gray; PB, parabrachial nucleus; EWcp, centrally-projecting Edinger-Westphal nucleus; NTS, nucleus of the solitary tract; LC, locus coeruleus; BBB, blood brain barrier; IC, insular cortex; PVN, hypothalamic paraventricular nucleus; BNST, bed nucleus of the stria terminalis; central (CeA) and medial (MeA) nucleus of the amygdala; NA, nucleus accumbens; VTA, ventral tegmental area; mPFC, medial prefrontal cortex; ACC, anterior cingulate cortex.

The seminal gate control theory of pain shifted pain research from the Cartesian view of the brain as a passive receiver of pain signals presumed to be generated in damaged tissue to the current understanding of the central nervous system as the dynamic source of pain [65]. Melzack [66] further developed the idea of the centrality of pain by theorizing the “neuromatrix” as a neural network integrating sensory-discriminative (e.g., nociceptive-pain), affective-motivational, and evaluative-cognitive dimensions in the construction and embodiment of pain experience. Several decades of empirical research continues to strongly support this explanatory model and paradigm shift in pain research. Figure 1 shows several of the components of the pain system identified in this review as it relates to Melzack’s conceptualization of the “neuromatrix”.

Nociceptors are dorsal root ganglion neurons in the peripheral nervous system that project to the dorsal horn of the spinal cord with myelinated (A-Delta fibers) or unmyelinated (C-fibers) axons, synapsing with secondary neurons in Rexed laminae I and II of the gray matter (also known as the marginal zone and substantia gelatinosa, respectively) and connecting with interneurons, descending modulating neurons, and afferent neurons in other laminae. The neurotransmitters involved in excitatory interactions include glutamate and substance P, while inhibitory neurotransmitters include GABA. The secondary neurons cross the midline and project to supraspinal structures via two primary paths through the thalamus as part of the anterolateral system (except for nociceptors of the face which follows a separate route to the thalamus via the trigeminal nerve). The sensory-discriminative dimension (e.g., nociceptive-pain) is attributed to the lateral pain system which includes the spinothalamic tract carrying information to tertiary neurons in the lateral thalamic nuclei that project to the posterior insular cortex and primary somatosensory cortices to provide information about location and intensity of nociceptive stimulation, while some ascending fibers make direct connections with the reticular formation in the brainstem (spinoreticulothalamic tract), possibly to direct attention to nociceptive stimuli. A separate medial route carries information to the periaqueductal gray (PAG) and the parabrachial nucleus (PB) at the junction of the pons and midbrain in route to the amygdala and other forebrain structures attributed to the affective-motivational and evaluative-cognitive dimensions of the neuromatrix [67, 68]. Interestingly, experiments with decerebrate animals which remove the integration of forebrain structures with the hindbrain by surgical separation of the connection with the brainstem and spinal cord have demonstrated intact escape-like behaviors (i.e., nocifensive behaviors) to specific noxious stimuli [69]. The PB is one critical structure receiving nociceptive input that appears to diverge into at least two distinct pathways. One neural pathway for the direct activation of nocifensive behavior (via the ventromedial hypothalamus or lateral PAG) and another pathway for the experience of pain and learning involving the forebrain structures, bed nucleus of the stria terminalis (BNST) or central nucleus of the amygdala (CeA) [70]. Thus, ascending nociceptive information (along with descending modulating influences) is integrated at many levels of the neuroaxis resulting in neural pathways that mediate many nociception-related functions - from the activation of nocifensive behaviors to the integration of nociceptive information with affect, emotion, cognition and learning [71]. It is the latter integrative function that transforms nociceptive information from a basic sensory experience (Melzack’s sensory-discrimination dimension) to a constructed perception that is experienced as pain [66, 72].

## Supraspinal structures involved in affective-motivational aspects of pain

Supraspinal pain pathways include complex circuitry classically associated with affect/emotion^2^ and reward which are also critical contributors to drug and alcohol misuse and dependence according to some extant theoretical models of addiction (see [73]). These shared circuits may also explain why world-wide across many cultures the same words (e.g., “pain” and “hurt”) are often used to describe seemingly different experiences such as actual physical injury (i.e., nociceptive-pain) and rejection from a social partner (social or psychological pain) [74]. In one functional MRI study the experimental induction of nociceptive-pain and social pain in the same participants activated the same cortical structures indicating that the two negative emotions shared similar somatosensory representations [75]. Other functional imaging studies provide support for the similarity and shared forebrain structures between nociceptive-pain and social pain [76]. Among the forebrain structures shared by these is most notably the amygdala and its various inputs and outputs.

The amygdala—consisting of 3 nuclear groups, the basolateral amygdaloid complex (BLA), central nucleus (CeA), and cortical-like group (Co)—is well suited for the integration of sensory/perceptual and affective/emotional information. The main subregion receiving extrinsic (sensory) information, including nociceptive information, is the BLA group which consists of the lateral nucleus (LA), basolateral nucleus (BL), and basomedial nucleus (BM). The LA is the primary input region receiving projections from higher order sensory association areas of the cortex and connecting reciprocally with the other BLA nuclei and other amygdala groups [77, 78]. The BLA also receives projections from the medial prefrontal cortex (mPFC) which provides affective (or valence) information and emotion-based information modulated by executive functions (e.g., decision making) to guide behavior and action [79]. The primary neurotransmitters in the amygdala circuitry is glutamate involving excitatory transmission and GABA involving tonic and phasic inhibitory transmission [80]. The major output of the amygdala complex is the CeA which has extensive projections to the lateral hypothalamus, basal forebrain regions, and brainstem. The CeA also forms a circuit referred to as the *extended amygdala* hypothesized to be involved in evaluating the affective value of sensory stimuli.

The concept of the *extended amygdala* was first introduced a century ago when comparative developmental neuroanatomy studies determined that the CeA forms a continuous pathway with the BNST in vertebrates [81]. Extensive experimental studies demonstrating the key role of the extended amygdala in the acquisition and expression of fear memories has established these structures as key components of an emotion brain circuit [82]. In adult mammals the extended amygdala consists of the BNST, central and medial amygdalae (CeA, MeA), and a transition zone in the shell (medial portion) of the nucleus accumbens (NA). These structures are not only important in emotion, but are also involved in learning, memory and reward processes that allow emotion to be integrated with perception, learning, memory, and behavioral action. The recruitment of the extended amygdala is hypothesized to play an important role in the multistage model of alcohol dependence [73]. With respect to pain, structures such as the CeA serve to integrate nociceptive information and modulate the perception of pain through its outputs to the forebrain, brainstem and spinal cord. Most of the brain pathways associated with pain have been elucidated using rodents primarily, but with support from human neuroimaging studies. Correlations between amygdala activity and pain-like responses in rodents, and pain verbal reports in people, have been widely reported [50, 79, 83, 84]. The involvement of the CeA neurons in nociceptive and pain-related processing possibly via input from the paraventricular nucleus of the thalamus, has been described as the “nociceptive amygdala” [85]. The CeA has also been described as an “integrative hub” for negative affect (e.g., anxiety) and alcohol use disorders [80]. Nociceptors project axons to the CeA through the parabrachial (PB) nucleus providing information about a range of homeostatic functions including information about noxious stimulation [86]. The PB also receives “top-down” descending pain modulatory signals [87]. Dysregulation of excitatory and inhibitory neural activity may result in neuropathic pain as suggested by optogenetic studies with mice experiencing experimentally-induced hypersensitivity to aversive stimuli [88]. For example, hyperalgesia induced by alcohol withdrawal in alcohol-dependent rats is mediated by CeA projections to the ventrolateral PAG neurons containing µ-opioid receptors. The CeA distributes GABAergic neurons to these PAG neurons to inhibit the perception of pain, but in rats experiencing withdrawal in an alcohol vapor model of alcohol dependence the inhibitory CeA signals were weakened thereby facilitating nociception signals and likely leading to increased nociceptive-pain (i.e., hyperalgesia) [89]. Recent work also implicates changes in dopamine-, melanocortin- and corticotropin-releasing factor signaling in the reciprocal relationship between the midbrain and CeA that may be moderated by sex and age [90].

### Affective-emotional brain structures and alcohol

A role for several of these same forebrain structures in alcohol consumption was first implicated by Chang et al [91] when rats treated with intraperitoneal injections of alcohol showed dose-dependent increases in the immediate early gene, c-FOS, activation (a marker of neuronal activity) in the PB, BNST, and CeA as well as the Edinger-Westphal nucleus (EW), paraventricular hypothalamic nucleus (PVN), and locus coeruleus nucleus (LC).

The Chang et al [91] study was complemented by the finding that alcohol is as effective as LiCl to induce conditioned taste aversion and an associated increase of FOS expression in the PB [92].^3^ The PB is now well known to be a crucial structure for conditioned taste aversion - an important learned behavioral strategy to defend homeostasis by avoiding subsequent exposure to previously consumed life-threatening substances [93, 94]. Similar alcohol-induced FOS expression have been found in different mouse strains genetically selected to engage in high levels of alcohol self-administered while engaged in different patterns of intake, although strain differences in c-FOS activation were observed in other brain regions associated with ethanol drinking [95]. While these FOS immununoreactivity studies confirmed that the PB at the very least receives information concerning the presence of aversive systemic alcohol, subsequent studies demonstrated a role of the PB in modulating alcohol consumption. For example, optogenetic stimulation of neurotensin neurons projecting from the amygdala to the PB increases the intake and rewarding value of alcohol and other palatable solutions [96].

These findings indicate that the PB is involved in the aversive and rewarding properties of alcohol. Although alcohol exposure (by experimental treatment or self-administration procedures) is initially aversive, the aversive properties decline with repeated exposure to ethanol and the rewarding properties increase. Indeed, c-FOS activation following acute ethanol administration causes c-FOS activation to decline (desensitize) in the PB and other alcohol-sensitive brain structures at different rates with the EW showing more sustained sensitivity than the other nuclei [91].

The earliest studies demonstrating sensitivity to alcohol in the Edinger-Westphal nucleus in the brain stem was surprising because this structure was known to be a part of the oculomotor nuclear complex sending parasympathetic nerve fibers to the eye. However, the structure is now recognized to consist of distinct brain regions, a preganglionic EW nucleus projecting to the ciliary ganglion to regulate oculomotor function and a centrally-projecting nucleus (EWcp) that is highly sensitive to alcohol administration and projects to several brain regions including the BNST, CeA, dorsal raphe nucleus (DRN), anterior cingulate cortex (ACC), preoptic area (POA), medial prefrontal cortex (mPFC), lateral hypothalamus (LH), and ventral tegmental area (VTA). The EWcp neurons express several neuropeptides known to be associated with stress, reward, and administration of drugs of misuse, including urocortin 1 (Un1), cocaine and amphetamine-regulated transcript (CART) and substance P [97, 98]. There is also evidence from animal studies that the EWcp is activated in response to nociceptive stimuli and is possibly involved in chronic pain. A cluster of EW neurons with colocalized cholecystokinin (CCK) and substance-p in rats increases its firing rate in response to nociceptive simulation (toe-pinch). This neuronal activation is suppressed by systemically administered morphine—an effect reversed by naloxone [99]. Noxious visceral stimulation of the EW in rats increased expression of immediate early genes including c-FOS [100].

Interest in the role of EW in stress, pain, and alcohol consumption increased with the discovery of Un1 neurons [97]. Un1 belongs to the CRF neuropeptide superfamily, the principal hypothalamic stress-related neuropeptide, and binds with CRF-1 and CRF-2 receptors to induce G-protein-coupled signaling. The EWcp has the largest population of Un1 neurons often colocalized with CART. The EWcp projects to many sympathetic-innervated targets in the brainstem and spinal cord and has been proposed to function as a central orchestrator of the sympathetic nervous system’s response to stress [97].

Interestingly, pain, stress, and alcohol induce a delayed and more sustained neural activation in the EW compared to other brain nuclei. As a comparison, for example, corticotropin releasing factor (CRF) releasing neurons in the PVN show more immediate and transient activation following an acute stressor stimuli [101]. Sustained activation of Un1 neurons also occurs following acute formalin-induced nociceptive-pain and chronic ether stress [97, 102]. These and other findings have led to the hypothesis the EWcp plays a critical role in adapting to bodily perturbations caused by acute stressful events, physical injury (nociceptive-pain), and ingestion (including potentially dangerous compounds such as alcohol) [97, 101, 103]. Thus, the EWcp is a likely key player in energy metabolism and the defense of homeostasis (for review see [101]). However, chronic stress, repeated pain experiences, and any associated alcohol and drug use may disrupt the return to homeostasis causing an allostatic shift (i.e., the establishment of a new homeostatic state) and the emergence of enduring, relapsing conditions such as chronic pain or the behavioral changes seen in the addiction phenotype [101, 104].

## Homeostasis and allostasis

Occasional acute physical disturbances or infrequent experiences that may be a potential threat (stressor) result in an adaptive protective response followed by the return to a static but “normal” homeostatic function. Homeostasis makes sense within a physical system that maintains stable features to match an environment that is unchanging notwithstanding irregular and temporary perturbations. However, with the emergence of a chronic environmental stressor or persistent repeated exposures to physical insults the maintenance of a “normal” homeostatic baseline no longer makes sense. To adapt to these new persistent environmental demands allostatic processes are engaged that predict the optimal physiological parameters needed to achieve stability [105]. Thus, unlike homeostasis which maintains optimal parameters within steady state “normal” levels, allostasis is a dynamic whole-body process involving the prediction of optimal levels of functioning based on anticipated demand from changing environmental variables. In essence, the body is learning to adapt to changing environmental demands. Although allostasis reflects efficient physiological regulation, current allostatic models of disease conceptualize the gradual life-time buildup of “wear and tear” of the body (or allostatic load) as causing the overactivation or dysregulation of allostatic systems that mediate the effects of chronic stress on disease and mental health [105, 106]. This concept of maladaptive allostasis in brain stress systems have also been advocated to explain addiction and possibly chronic pain.

According to the multistage model of the development and maintenance of alcohol addiction proposed by Koob et al., stress and reward systems undergo changes to maintain hedonic stability in an allostatic state [73, 104, 107]. However, the buildup of allostatic load may progress an individual towards alcohol misuse and addiction in 3 stages of motivated behavioral change: 1) binge and intoxication driven by positive reinforcement, 2) withdrawal and negative affect relieved through negative reinforcement, and 3) preoccupation and anticipation with the drug of choice that is mediated by associative learning (i.e., Pavlovian conditioning).

Maladaptive allostasis in addiction emphasizes the role of emotional states in guiding motivated behavior. Initially alcohol may provide a pleasant affective/emotional experience. It may be positive reinforcement (or reward) due to the pleasant experience of the alcohol consumption (“a buzz”) or the social approval of drinking in the presence of others. Or it may be negative reinforcement as a result of the temporary reduction of an unpleasant experience such as transient relief of physical or psychological pain. In either case the exogenously administered alcohol induces a departure from homeostasis and thus the body will address the temporary alcohol-induced perturbations (no matter if the effects are willfully wanted or unwanted by the individual) by activating opponent-like processes that counteracts the drug as well as the concomitant affective/emotional change [104, 108]. According to an allostatic perspective repeated exposure to alcohol intake (interacting with genetic factors, unique life experiences and psychiatric co-morbidities) can result in maladaptive allostasis leading to pathological states such as alcohol dependence. Koob proposed the psychological construct of hyperkatifeia, an exaggerated negative emotional state (i.e., increased psychological pain and distress) that can occur during periods of alcohol withdrawal to maintain addictive behavior through craving and negative reinforcement [63]. This heightened emotional state has a parallel in the pain system in the form of the transition from alcohol-induced analgesia to alcohol-induced hyperalgesia and chronic pain [109].

## Self-medication with alcohol

Before discussing self-medication with alcohol, it is worth nothing that acute and chronic consumption of alcohol has many potential injurious effects on the body. The largest area of investigation has been on the role of chronic alcohol misuse on the burden of preventable diseases of the liver, pancreas, and gastrointestinal tract [110]. Clinical studies and preclinical models indicate that females experience greater harms from alcohol despite drinking less than males, yet the gap in alcohol consumption between men and women is now narrowing (e.g., [111–114]). Some of the deleterious effects of chronic alcohol misuse and addiction are due to consequent nutritional deficiencies. Chronic alcohol consumption often leads to reduced intake of dietary thiamine (Vitamin B1) which is further exacerbated by alcohol-induced malabsorption of this essential vitamin. Thiamine deficiency interferes with several critical cellular functions resulting in toxic effects on several brain regions leading to disorders of the brain such as Wernicke-Korsakoff syndrome and to neuropathies of the peripheral nervous system [115]. More direct mechanisms of neuropathic pain caused by alcohol or its metabolites have been proposed and are active areas of investigation; for example, oxidative stress nerve damage due to overproduction of ROS, sustained activation of the hypothalamic-pituitary-adrenal (HPA) axis, overactivation of protein kinase C (PKC), and dysregulated neurocircuitry are just a few examples of possible mechanisms [116]. Excessive misuse of alcohol is also causally associated with neurodegenerative disorders (e.g., Huntington’s disease, generalized dementia, multiple sclerosis) and some types of cancers (e.g., upper alimentary tract and liver) [117, 118]. Acute effects of alcohol can also induce different degrees of injurious outcomes. Research implicates neuroinflammation involving TLR4 and TRPV1 in the transient effects of alcohol-induced headaches experienced by some people when drinking fermented beverages [119]. Acute but excessive amounts of alcohol may also interfere with the innate immune system defense against bacterial infection by injuring hematopoietic tissue and impairing bone marrow production of granylocytes (including neutrophils, eosinophils, and basophils) increasing vulnerably to bacterial infection and sepsis [120]. And, of course, intoxicating levels of alcohol increases vulnerability to engage in risky behaviors that can result in highly injurious outcomes leading to long-term pain and disability as well as loss of life. In a recent meta-analysis, 27% of fatalities from non-traffic injuries were attributable to misuse of alcohol [121].

Notwithstanding our current knowledge of alcohol misuse as a leading risk factor for disease burden, since antiquity there has been an enduring belief in the medicinal power of alcohol. Evidence indicating a complex association between alcohol use and health includes several decades of evidence for the protective benefits of moderate alcohol use on cardiometabolic health, for example, [122, 123]. More recently, there has been an increase in caution expressed about the view that alcohol-in-moderation yields health benefits. Despite the promising results of many short-term randomized controlled studies, this concern over the presumed health benefits of alcohol is based on the lack of long-term randomized trials of moderate alcohol consumption compared with no (or very low) alcohol drinking [124]. As a result, interest in Mendelian randomization (MR) studies has grown in popularity. MR is an epidemiological method that mimics a randomized long-term controlled setting to establish possible causal relationships in observational data [125]. In conventional observational studies the presence of a causal relation between alcohol consumption (a potential cause) and a protective health outcome is limited by the possible presence of confounding variables, reverse causation, and measurement error. In a Mendelian randomization design genetic variants (e.g., ALDH2 polymorphic gene) that are reliably associated with different levels of exposure to a potential causal factor (e.g., alcohol consumption) but uncorrelated with the outcome of interest (e.g., cardiovascular disease) is analyzed to estimate a true causal effect between the potential causal factor and the outcome (if any). However, MR studies that have investigated the effects of alcohol drinking on cardiovascular health have been inconsistent suggesting that further studies are needed for refinement of MR and integration with other research methods [125–129]. Nevertheless, there is substantial evidence in humans and rodents that acute consumption of alcohol can be motivated by the experience of efficacious self-medication. The self-medication hypothesis is a causal model that posits that individuals drink alcohol under aversive conditions as a way to cope with anxiety, depression, and pain (i.e., negative reinforcement).

Support for the self-medication model comes primarily from studies investigating self-medication as a contributor to abusive alcohol use comorbid with anxiety and depressive disorders [130–133]. That is, as drug or alcohol use becomes a more frequently relied upon as an efficacious coping strategy, the use can transition to problematic use and addiction. Laboratory experiments also demonstrate the effectiveness of alcohol consumption in reducing experimentally induced stress, although these effects may rely on the influence of prior experience and the type of stressor [134, 135]. Preclinical studies demonstrate that rodents will self-medicate with alcohol and some anxiolytics when experiencing aversive emotional states (psychological pain) induced by loss or reduction of expected reward. For example, rats show a greater consumption of alcohol over water immediately after an expected highly preferred reward is omitted or reduced to a less preferred value [136–138]. Interestingly, reward loss also induces a reduced sensitivity to nociceptive-pain (hypoalgesia) which appears to reflect activation of a compensatory opioid and cannabinoid system to modulate physical and psychological pain as a component of homeostatic and allostatic modifications [74]. It is clear that low or moderate amounts of consumed alcohol also exerts clinically relevant hypoalgesic effects in controlled experimental studies with people and animals [50, 55, 56, 139]. Similar effects of alcohol and endogenous opioids on nociceptive-pain suggest an intersection of neural circuits, more specifically the opioid-mediated regulation of GABA neurotransmission [109, 140]. The possible involvement of alcohol’s effect on inflammation and inflammatory cytokines acting on µ-opioid receptor regulation also needs further investigation [141].

### Hypoalgesia and hyperalgesia

Paradoxically, while acute alcohol drinking reduces sensitivity to pain repeated administration of alcohol, like opioids and other analgesic drugs, results in greater sensitivity to physical nociceptive-pain-inducing stimuli (hyperalgesia). Evidence of opioid-induced hyperalgesia after chronic exposure to opioids is well established in preclinical studies and is observed in clinical populations particularly individuals with opioid use disorder [124, 142]. Chronic alcohol consumption results in neural alterations that are also seen in chronic pain—a decrease in inhibitory GABA activity along with hyperglutamatergic activity [109, 143, 144].

The transition from efficacious reduction in psychological and physical pain during acute alcohol administration to the opponent-like process of hyperalgesia appears to be exacerbated with repeated experiences of withdrawal from alcohol. Chronic voluntary alcohol consumption induces hyperalgesia in rats, an effect that further increases during periods of alcohol withdrawal [145, 146]. A recent experimental study also demonstrated alcohol withdrawal-associated hyperalgesia in young adult binge drinkers with only 1–3 years of drinking history [147].

The effects of alcohol withdrawal in animal models are particularly interesting. Early models of AUD required the time-consuming procedures to induce pharmacologically-relevant levels of alcohol in rodents and primates such as sucrose-fading procedure and scheduled-induced polydipsia. For example, sucrose-fading procedure exposed rats to mixtures of ethanol and sucrose to drive high levels of consumption followed by the gradual reduction of sucrose to zero [148, 149]. Many current studies use intermittent access to unadulterated alcohol often in binge-like patterns to elevate consumption in rodents (relative to continuous access). One possible mechanism of escalated drinking in intermittent procedures appears to be repeated periods of acute withdrawal, which would be accompanied with withdrawal-induced hyperalgesia and other aversive experiences. Interestingly, alterations in glutamate neurotransmission are consistently associated with intermittent procedures compared to rodents continuously exposed to alcohol [150].

## Top-down construction and modulation of pain perception

It is well established in the field of perception that the experience of perception in any modality is influenced by top-down cognitive processes determined by context or expectations and beliefs based on prior experiences. This includes pain perception. As already discussed, it is inadequate to view pain as a direct readout of nociceptive input. Early theories explaining pain in terms of direct dedicated pathways for nociception began to be questioned by paradoxical observations such as the observation of less than severe pain or no pain in soldiers with extensive wounds [151]. The phenomena of phantom limb (persistent sensations in a missing or amputated limb) and placebo hypoalgesia (pain relief from the expectation of a beneficial or therapeutic outcome) inspired Melzack to include the evaluative-cognitive dimension in the neuromatrix theory of pain perception [66]. Brain structures implicated in the cognitive modulation of pain include the anterior insular cortex (IC) and anterior cingulate cortex (ACC), structures shared with circuitry implicated in emotion, reward, and drug and alcohol addiction [73, 152]. The PAG along the caudal rostral axis of the midbrain is the most well-characterized pathway involved in descending pain modulation through its connection with the dorsolateral PFC, rostral ACC, hypothalamus, and ventromedial medulla, and spinal cord [71, 153]. Experimental human studies on placebo hypoalgesia and expectation effects show that the descending modulation of pain pathways are mediated primarily through endogenous opioids and dopaminergic signaling mediating negatively reinforcing pain relief or expectations of pain persistence, for example [154–156].

Placebo reduction of nociceptive processing at the level of the spinal cord shows the role of cognition in modulating nociceptive-pain at the level of sensory-discrimination dimension [153]. However, cognitive factors likely play more than a modulatory role in pain perception. As seen in other sensory modalities, top-down processing is fundamental to the construction of percepts resulting in individual differences in perceptions of the external world as revealed by ambiguous stimuli. As an example, consider the image of “the dress” that took the internet by storm in 2015 generating substantial interest among the public and the vision science community. Some observers perceived an overexposed image of a dress as black and blue—the actual color of the dress—while to others the dress appeared gold and white. People debating the dress color were incredulous—how can others looking at the identical image see colors that were undoubtedly wrong as informed by their own eyes? One possible explanation came from an empirical study showing that the ambiguous nature of the image required spontaneous assumptions about the source of lighting in the image for disambiguation, assumptions that differed depending on an individuals’ prior life experiences [157]. Similarly nociceptive-related signals, possibly emerging at multiple levels of the pain system, and homeostatic/allostatic feedback may sometimes be ambiguous to the pain system. The uncertainty of pain and other harmless bodily sensations (see [158]) may be disambiguated by the individual’s prior life experiences, expectations, and beliefs resulting in divergent interpretations and idiosyncratic experiences of pain. Contemporary perception research provides guidance on how to approach verbal reports of acute or chronic pain in the absence of evidence of tissue damage. Rather than dismissing such reports as “all in their heads” they should be treated as no less real than any other percept. The extent to which alcohol use, misuse and addiction contributes to ambiguity and disambiguation in the pain system and integrative neural networks may be a fertile area for investigation.

## Discussion

In this narrative review, we aimed to present an overview of the current understanding of the mechanisms of nociception, the sensation of nociceptive-pain, and pain perception to inform and guide research on the contribution of the pain system in alcohol use, misuse, and dependence. Conventional wisdom influenced by the centuries-old Cartesian model of pain views physical hurt as a nociceptive experience that is directly translated into the sensation and perception of pain. However, it has become clear that nociception and pain are closely related but distinct mechanisms of homeostasis in defense against injury and potential injury. It will be useful to investigate the impact of alcohol use and misuse on the role of nociception in the direct defense against noxious stimuli and pathogenic microbes through its action on the innate and acquired immune system and bidirectional neuroimmune communication. Such studies are likely to provide insight into how these alcohol effects influence the sensation of nociceptive-pain and possibly how alcohol-induced effects impact on bottom-up inputs for the constructive perception of pain. The influence of alcohol use on nociceptive processes and nociceptive-pain may provide a better understanding of the paradoxical effects of repeated alcohol use such as the transition from alcohol-induced hypoalgesia to alcohol-induced hyperalgesia. The excessive use of alcohol may contribute to additional changes in the pain system resulting in the development of chronic pain and maintenance of the abusive alcohol use behavior by negative reinforcement processes perhaps further mediated by maladaptive homeostasis and allostasis, contributing to progression and maintenance of addiction (i.e., alcohol use disorder).

Brain structures involved in neural pain circuits are shared with pathways mediating emotion and reward, as well as neural circuits that play a role in psychological disorders associated with stress, fear, anxiety, depression, and drug and alcohol misuse and dependence. The complex interrelationship between neuroimmune interactions and the neural circuits and networks involved in negative emotion, pain, and drug use disorders suggest that the activation of pain circuitry may play a role in the development and maintenance of alcohol dependence.

The role of reinforcement processes and top-down cognitive processes in the construction and modulation of pain perception (as well as gender-specific differences) validates the importance of identifying and establishing psychological approaches to prevent the transition of acute pain towards chronic pain, alcohol misuse, and alcohol addiction.

## Footnotes

1 The terms analgesia and antinociception are often used synonymously with hypoalgesia, however the former is more appropriately defined as the absence of pain in response to a stimulus that would otherwise be subjectively experienced as painful whereas hypoalgesia and antinociception refer to diminished pain.

2 Affect, mood, and emotion are subjective terms that are not consistently differentiated from one another. Affect is more typically defined as a broad range of subjective experiences that vary in terms of valence (positive to negative) and level of arousal. Emotion and mood are considered distinct phenomena, with the former typically short in duration and directed at a stimulus source.

3 Lithium chloride (LiCl) is a compound commonly used to establish conditioned taste aversions in preclinical studies using animals.
